# The correlation between tic disorders and allergic conditions in children: A systematic review and meta-analysis of observational studies

**DOI:** 10.3389/fped.2023.1064001

**Published:** 2023-03-20

**Authors:** Ying Chang, Ying Zhang, Yifan Bai, Run Lin, Yaping Qi, Min Li

**Affiliations:** ^1^ Department of Traditional Chinese Medicine, Children’s Hospital Capital Institute of Pediatrics, Beijing, China; ^2^ Evidence Based Medicine Center, Beijing University of Chinese Medicine, Beijing, China; ^3^ Department of Pediatrics, Beijing Hospital of Traditional Chinese Medicine, Capital Medical University, Beijing, China; ^4^ College of Traditional Chinese Medicine, Beijing University of Chinese Medicine, Beijing, China; ^5^ TCM Pediatrics Department, Beijing Changping District Maternity and Child Care Hospital, Beijing, China

**Keywords:** tic disorders, allergic conditions, systematic review, meta - analysis, children

## Abstract

**Aim:**

To evaluate the correlation between tic disorders and allergies and to inform strategies for the treatment and prevention of tic disorders.

**Methods:**

We conducted online searches of the MEDLINE, Embase, Cochrane, CNKI, CBM, WanFang, and VIP Information databases. Case-control studies and cohort studies related to tic disorders and allergic conditions were searched. Two researchers screened the literature, extracted data, and evaluated quality in strict accordance with the predetermined retrieval strategy and inclusion criteria. Finally, RevMan 5.4 software was used to conduct a meta-analysis. We used the Grading of Recommendations, Assessment, Development, and Evaluations (GRADE) approach to rating the certainty of evidence about each allergy outcome as high, moderate, low, or very low.

**Results:**

We obtained seven eligible studies involving eight allergic conditions. The following allergic conditions were significantly associated with the presence of a tic disorder: asthma (OR = 1.90, 95% CI = 1.57–2.30, *P* < 0.001), allergic rhinitis (OR = 2.61, 95% CI = 1.90–3.57, *P* < 0.001), allergic conjunctivitis (OR = 3.65, 95% CI = 1.53–8,67, *P* = 0.003), eczema (OR = 3.87, 95% CI = 2.24–6.67, *P* < 0.001) and food allergy (OR = 2.79, 95% CI = 1.56–4.99, *P* < 0.001). There was no significant correlation between atopic dermatitis, urticaria, drug allergy, and tic disorder.

**Conclusion:**

The occurrence of tic disorders may be associated with the presence of certain allergic disorders. However, whether allergy is one of the causes of tic disorders remains unclear.

**Systematic review registration:**

The registration number for this systematic review is PROSPERO: CRD42021231658.

## 1. Background

A tic is a sudden, rapid, recurrent, nonrhythmic motor movement or vocalization. Tic disorders are neuropsychiatric disorders that start in childhood and adolescence, with motor tics or vocal tics as the main clinical manifestations. The clinical manifestations usually include blinking, clearing of the voice, making faces, whole-body tics, and accompanying sounds and echoes. Tic disorders comprise three clinical subtypes: Tourette syndrome, persistent (chronic) motor or vocal tic disorder, and transient tic disorder (1, p. 947). In recent years, the incidence of tic disorders has been increasing worldwide. A systematic review and meta-analysis published in 2012 estimated the prevalence of Tourette syndrome at 0.77% and that of transient tic disorder at 2.99%, with boys being more commonly affected. The tic disorder prevalence in South Korea increased from 0.19% to 0.29% between 2009 and 2016 (2, p. 77–90; 3, p. 764–772). At present, due to insufficient knowledge about tic disorders, children with tic disorders are often alienated from their peers in daily life. Studies have shown that depression is a significant feature of Tourette syndrome, and individuals with tic disorders have an increased risk of both suicide death and attempted suicide (4, p. 127–139; 5, p. 111–118; 6, p. 128–132). For these reasons and others, research on tic disorders is vital.

At present, the etiology and pathogenesis of tic disorders in children are not entirely clear, but these disorders are generally believed to be related to genetic, environmental, psychological, and immunologic factors (7). Immune-related tic disorder was first reported in a 1985 case series by Finegold, who found elevated serum IgE levels and positive skin tests in all four Tourette syndrome patients; the report concluded that symptoms of tic disorders may be similar to allergies or co-existing with allergic conditions (8, p. 119–121). Clinically, we have found that many patients with tic disorders also suffer from allergies. Moreover, their tic symptoms tend to improve after they are treated for their allergies. However, our literature search suggested that the correlation between tic disorders and allergic disease is still controversial; there were discrepancies between the results of previous related studies. Therefore, we retrieved literature published before 2022-6-30 and obtained eight articles that met our predetermined requirements. Systematic evaluation and meta-analysis provided an expanded sample size to evaluate the correlation between the occurrence of tic disorders and allergic conditions. To provide a basis for the diagnosis, treatment, and prevention of tic disorders, we report the process and results of this review according to the PRISMA statement.

## 2. Methods

The protocol for this review was registered in PROSPERO (CRD42021231658) in February 2021.

### 2.1. Data sources and searches

We developed a detailed search strategy under the guidance of experienced search experts. We searched the MEDLINE, Embase, Cochrane, CNKI, CBM, WanFang, and VIP Information databases from inception through June 2022. We used both MeSH terms and free-form searches, such as “tic disorders” [MeSH], “tics” [MeSH], “Tourette syndrome”[MeSH], “Tourette*,” “tics*,” “asthma” [MeSH], “rhinitis, allergic” [MeSH], “conjunctivitis, allergic” [MeSH], “purpura, Henoch-Schoenlein” [MeSH], “eczema” [MeSH], “urticaria” [MeSH], “atopic dermatitides” [MeSH], “food hypersensitivity” [MeSH], “drug hypersensitivity” [MeSH]. The specific retrieval strategy can be viewed in the Supplementary Appendix S1.

### 2.2. Study selection

We included cohort studies and case-control studies that reported associations between tic disorders and allergic conditions, with participants aged 18 years or younger.

We excluded studies if their original data were not available.

After calibration exercises, teams of two reviewers independently screened titles and abstracts. Articles that either reviewer judged as potentially eligible then had full-text screening. Raters resolved disagreements by discussion or, if necessary, with a third reviewer.

### 2.3. Data extraction and quality assessment

We performed calibration exercises, and teams of two independent reviewers extracted data, addressed the risk of bias, and resolved discrepancies by discussion or consultation with a third reviewer. We used a predefined extraction form in Excel (Microsoft Corp., Redmond, WA, United States) for each study and included the following information: (1) basic information, such as the title, author, publication year, and study country; (2) basic characteristics of the research, such as the number of eligible participants at baseline, age and sex of participants, and types of tic disorders and allergic conditions; (3) key factors for evaluating the risk of bias; and (4) study endpoints, including the odds ratios (ORs), 95% confidence intervals (CIs), and the number of allergic conditions in tic and non-tic populations. If a study reported more than one adjusted OR, we selected the most adjusted value. If a study did not report ORs, we calculated ORs from frequency data. Table 1 summarizes the basic information of the included literature.

**Table 1:** Summary of the basic information of the included studies.

Study	Nation	Participants			Mean age		Boys %		Case	Exposure	Prevalence of allergic diseases		Risk of bias
		Total	Cases	Controls	Cases	Controls	Cases	Controls			Case	Control	
Chang 2011 (9, p. 98–102)	China	4,223	845	3,378	8.37 ± 2.97	8.38 ± 2.96	76.0%	Low risk	TS	Asthma	0.26	0.11	Low risk
										Allergic rhinitis	0.58	0.33	
										Allergic conjunctivitis	0.65	0.53	
										Atopic dermatitis	0.27	0.15	
Wu and Ji, 2014 (10, p. 7)	China	1,600	800	800	8.6 ± 2.59	8.85 ± 2.64	51.0%	High risk	TD	Asthma	0.15	0.09	High risk
										Allergic conjunctivitis	0.26	0.07	
Yuce 2014 (11, p. 303–310)	Turkey	67	32	35	12.7	10.6 ± 3.5	42.9%	Low risk	TS	Asthma	0.19	0.09	Low risk
										Allergic rhinitis	0.41	0.17	
										Eczema	0.09	0.06	
Shen 2017 (12)	China	120	60	60	/	/	76.7%	Low risk	TS	Allergic rhinitis	0.22	0.08	Low risk
Aksu 2020 (13, p. 141–147)	Turkey	180	79	101	11.4 ± 2.2	11.2 ± 2.2	83.2%	Low risk	TS	Asthma	0.11	0.05	Low risk
										Allergic rhinitis	0.29	0.20	
										Allergic conjunctivitis	0.22	0.14	
										Atopic dermatitis	0.19	0.13	
										Food allergy	0.14	0.04	
										Drug allergy	0.05	0.05	
Chen 2020 (14, p. 247–253)	China	140	70	70	6.82 ± 2.2	6.9 ± 2.3	71.4%	Low risk	TTD	Asthma	0.17	0.03	Low risk
										Allergic rhinitis	0.49	0.11	
										Allergic conjunctivitis	0.74	0.17	
										Atopic dermatitis	0.06	0.11	
Chang 2022 (15, p. 610–613)	China	432	216	216	8 (6–10)	8 (6–10)	79.2%	Low risk	TD	Asthma	0.08	0.04	Low risk
										Allergic rhinitis	0.62	0.35	
										Allergic conjunctivitis	0.34	0.09	
										Eczema	0.33	0.10	
										Urticaria	0.08	0.03	
										Atopic dermatitis	0.01	0.00	
										Food allergy	0.17	0.07	
										Drug allergy	0.01	0.00	

TD, tic disorder; TS, Tourette syndrome; TTD, transient tic disorder.

To address the risk of bias, we used a modified version of the Clinical Advances through Research and Information Technology (CLARITY) risk of bias tool (35). After resolving discrepancies, we classified items rated as “definitely low” and “probably low” as having a low risk of bias and those rated as “probably high” and “definitely high” as having a high risk of bias. We regarded all items as equally important and rated a study as having a high risk of bias if two or more items had a high risk of bias. Appendix 2 presents the risk of bias analysis of the included studies.

### 2.4. Data synthesis and analysis

All statistical analyses were done using RevMan, version 5.4 (The Cochrane Collaboration, London, United Kingdom), and different allergic conditions were analyzed. Using meta-analysis, we calculated the combined OR and 95% CI of the correlation of different allergic conditions with tic disorders. Heterogeneity among studies was examined by inspecting forest plots for overlapping CIs, *I*^2^ statistics, and Q statistics. Because of the heterogeneity of the study itself, random-effects modeling was adopted.

### 2.5. Certainty of evidence

We used the Grading of Recommendations, Assessment, Development, and Evaluations (GRADE) approach to rate the certainty of evidence of each allergy outcome as high, moderate, low, or very low (16, p. 924–926; 17, p. 995–8). One reviewer evaluated the certainty of the evidence, which was confirmed or revised by the senior reviewer. Evidence from observational studies begins at low certainty and may be increased to moderate or high certainty when a large effect is observed, when all plausible confounders and biases would reduce a demonstrated effect, or when a dose-response gradient is present. Observational studies may be downgraded to very low certainty because of study limitations, publication bias, inconsistency, imprecision, or indirectness.

## 3. Results

### 3.1. Study selection

**Figure 1:**
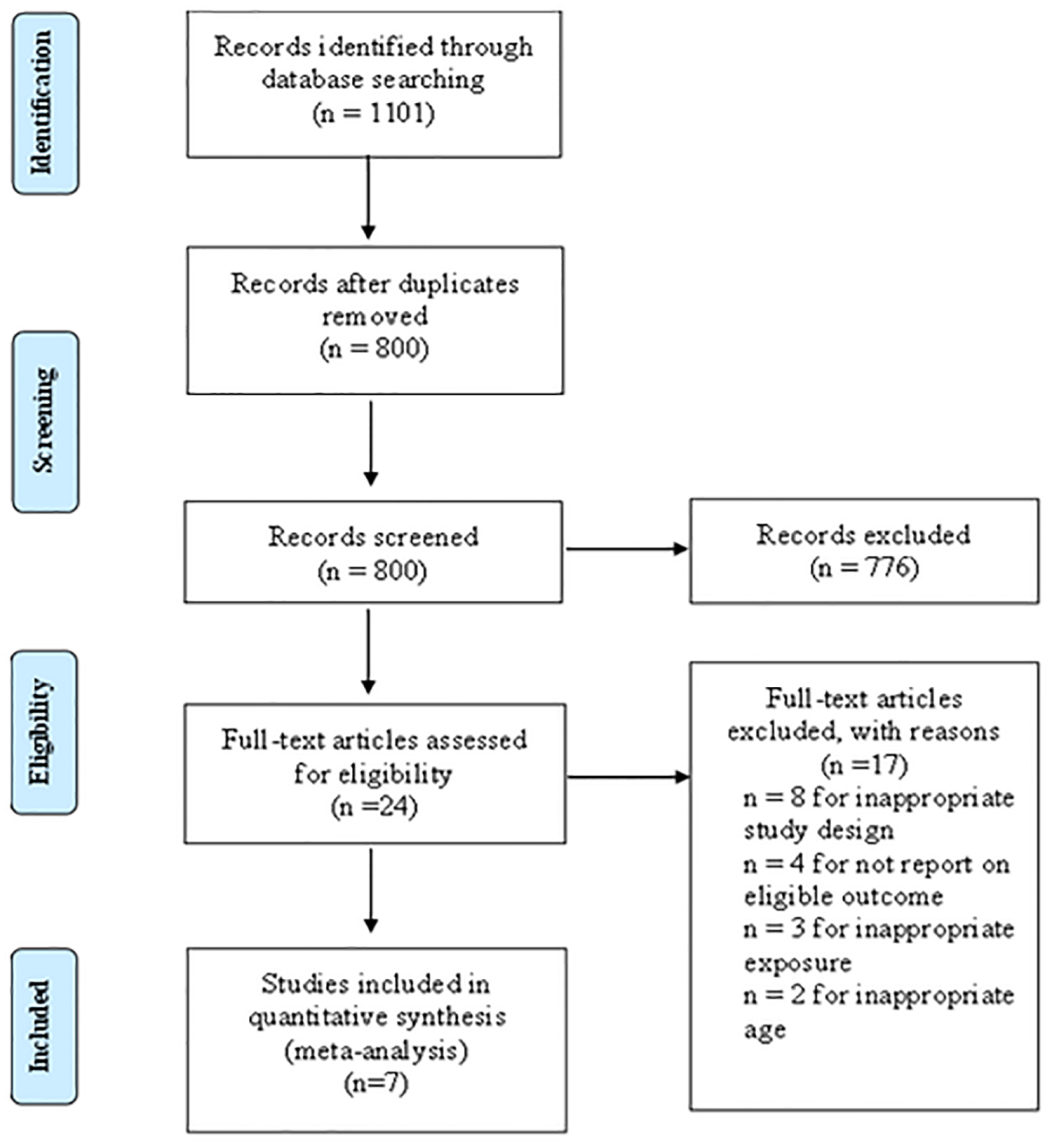
Study selection flow chart.

According to the predefined retrieval strategy (see Supplementary Appendix S1 for details), we retrieved a total of 1,101 articles, and 301 articles were excluded after deleting duplicate records. By browsing titles and abstracts, we selected 24 related articles and obtained the full texts. Eight articles were excluded because their research designs did not meet the requirements, four articles were excluded because the original data could not be retrieved, and three article was excluded because the exposure did not meet the requirements. Two articles were excluded because their age did not meet the requirements. Finally, seven articles met our requirements for this systematic review and meta-analysis. The study inclusion and exclusion flow chart is shown in Figure 1, and the basic characteristics of the final literature search are shown in Table 1.

### 3.2. Risk of bias

We used a scale to evaluate the quality of the included studies according to the established evaluation criteria. Among the seven included studies, one had high risk, and six had low risk. The evaluation results of different projects for each study are shown in Supplementary Appendix S2. The overall risk of bias assessment can be seen in Table 1.

### 3.3. Associations between tic disorders and allergic conditions

**Figure 2:**
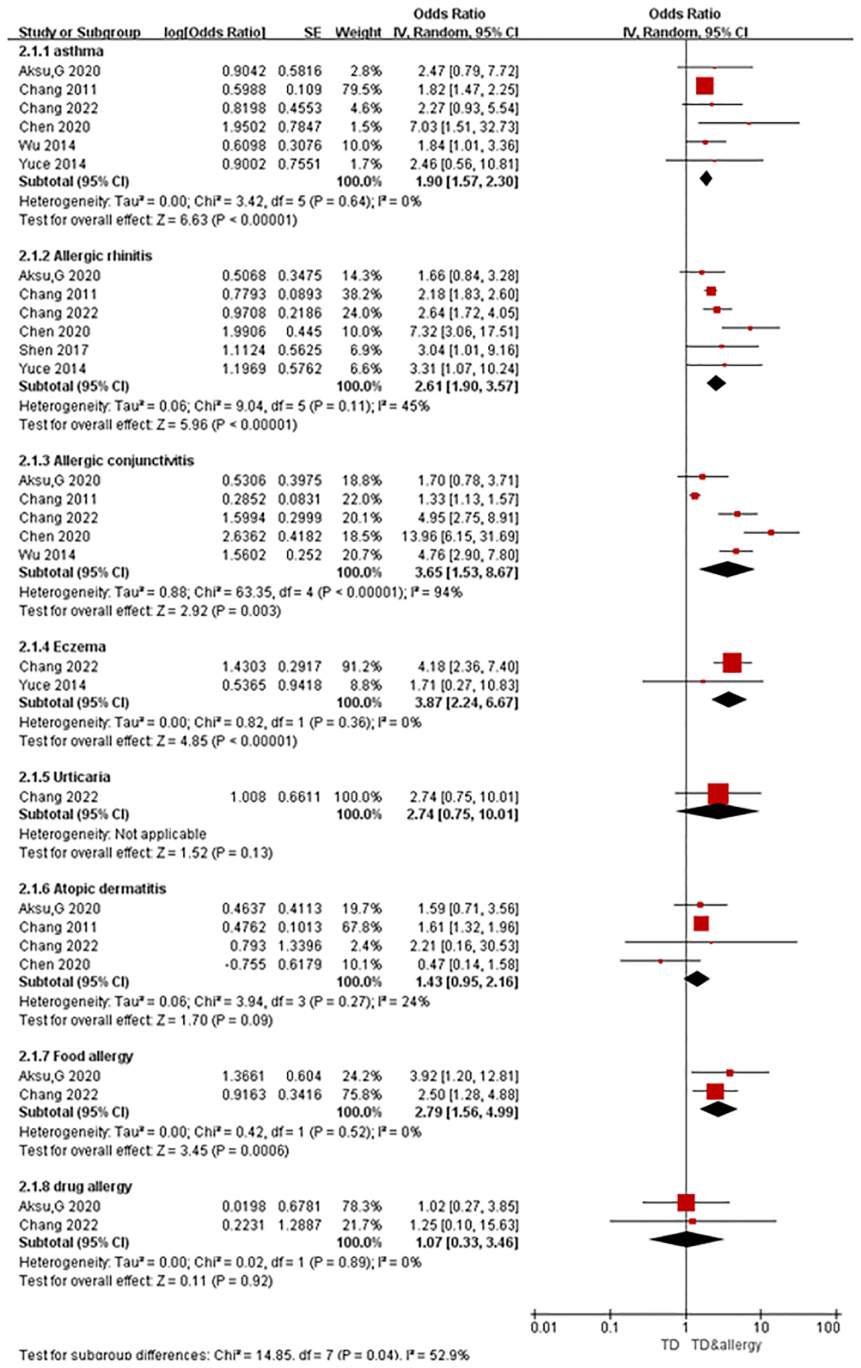
Forest map.

A total of seven articles were obtained, all of which were case-control studies involving eight kinds of allergic conditions, including six studies on asthma, six on allergic rhinitis, five on allergic conjunctivitis, two on eczema, one on urticaria, four on atopic dermatitis, two on food allergy, and two on drug allergy. Combined analysis was carried out according to the different allergic conditions, and the results of the random-effects model meta-analysis showed that among the eight different allergic conditions analyzed, five were significantly associated with tic disorders. As Figure 2 shows, the prevalence of asthma (OR = 1.90, 95% CI = 1.57–2.30, *P* < 0.001), allergic rhinitis (OR = 2.61, 95% CI = 1.90–3.57, *P* < 0.001), allergic conjunctivitis (OR = 3.65, 95% CI = 1.53–8,67, *P* = 0.003), eczema (OR = 3.87, 95% CI = 2.24–6.67, *P* < 0.001) and food allergy (OR = 2.79, 95% CI = 1.56–4.99, *P* < 0.001) was significantly higher in the cases (individuals with tic disorders) than in the control group. In contrast, the presence of atopic dermatitis (OR = 1.43, 95% CI = 0.95–2.16, *P* = 0.09), urticaria (OR = 2.74, 95% CI = 0.75–10.01, *P* = 0.13), and drug allergy (OR = 1.07,95% CI = 0.33–3.46, *P* = 0.92) were not significantly associated with the presence of tic disorders.

### 3.4. Certainty of evidence

All of the included studies were case-control studies, and none of the results exhibited serious study limitations or indirectness. The heterogeneity for outcomes of asthma, allergic rhinitis, eczema, urticaria, atopic dermatitis, food allergy, and drug allergy were relatively low, but severe inconsistency (*I*^2 ^= 94%) existed for allergic conjunctivitis. The outcomes of urticaria, atopic dermatitis and drug allergy exhibited serious imprecision (95% CIs contained invalid values). The outcome of urticaria had serious publication bias (inclusion of 1 study). The associations between either allergic rhinitis, allergic conjunctivitis, eczema or food allergic and tic disorders had a large effect size (OR > 2). According to the GRADE assessment, the evidence certainty for allergic rhinitis, eczema and food allergy were moderate, the certainty of evidence for asthma and allergic conjunctivitis were low, and the evidence certainty for other allergic diseases was very low. See Table 2 for more details.

**Table 2:** GRADE evidence profiles.

Outcome	Included studies	OR (95% CI)	Quality assessment					Rating up			Level of evidence
			Study limitations	Publication bias	Inconsistency	Imprecision	Indirectness	Large effect size	Dose-response gradient	Plausible confounding	
Asthma	(9–11, 13–15)	1.90 (1.57–2.30)	No	No	No	No	No	No	No	No	⊕⊕◯◯LOW
Allergic rhinitis	(9, 11–15)	2.61 (1.90–3.57)	No	No	No	No	No	Yes^a^	No	No	⊕⊕⊕◯MODERATE
Allergic conjunctivitis	(9, 10, 13–15)	3.65 (1.53–8.67)	No	No	Serious Inconsistency^e^	No	No	Yes^b^	No	No	⊕⊕◯◯LOW
Eczema	(11, 15)	3.87 (2.24–6.67)	No	No	No	No	No	Yes^c^	No	No	⊕⊕⊕◯MODERATE
Urticaria	(15)	2.74 (0.75–10.01)	No	Suspected^f^	No	Serious Imprecision^g^	No	No	No	No	⊕◯◯◯ VERY LOW
Atopic dermatitis	(9, 13–15)	1.43 (0.95–2.16)	No	No	No	Serious Imprecision^h^	No	No	No	No	⊕◯◯◯ VERY LOW
Food allergy	(13, 15)	2.79 (1.56–4.99)	No	No	No	No	No	Yes^d^	No	No	⊕⊕⊕◯MODERATE
Drug allergy	(13, 15)	1.07 (0.33–3.46)	No	No	No	Serious Imprecision^i^	No	No	No	No	⊕◯◯◯VERY LOW

CI, confidence interval; OR, odds ratio; GRADE, grading of recommendations assessment, development, and evaluation; ⊕⊕⊕⊕, high quality; ⊕⊕⊕◯, moderate quality; ⊕⊕◯◯, low quality; ⊕◯◯◯, very low quality.

^a–d^ ORs were 2.61, 3.65, 3.87 and 2.79 respectively.

^e^ High I square.

^f^ Only 1 study.

^g–i^ Recommendation would differ if the upper versus the lower boundary of the CI represented the truth.

## 4. Discussion

In recent years, the incidence of tic disorders has been rising worldwide. Tic disorders cause great distress to affected children and their parents. At present, the etiology and pathogenesis of tic disorders in children are not fully understood, but these disorders are usually considered to be related to genetic, environmental, psychological, and immune factors. Through vast clinical experience, we have observed that children with tic disorders tend to have concurrent allergic conditions, therefore, investigating the correlation between tic disorders and allergies may be helpful for the prevention and management for tic disorders.

### 4.1. Correlation between Td and allergic conditions

We systematically searched and evaluated studies that reported associations between tic disorders and allergic conditions. As a result, we included seven case-control studies in our meta-analysis. Six of these studies had a low risk of bias, one had a high risk of bias. The meta-analysis revealed that the allergic conditions associated with tic disorders were eczema (OR = 3.87), allergic conjunctivitis (OR = 3.65), food allergy (OR = 2.79), allergic rhinitis (OR = 2.61) and asthma (OR = 1.90). According to the GRADE assessment, the evidence certainty for allergic rhinitis, eczema and food allergy were medium, and the certainty of evidence for asthma and allergic conjunctivitis were low. There was no significant correlation between urticaria, atopic dermatitis, drug allergy, and tic disorder. The evidence certainty for urticaria, atopic dermatitis and drug allergy were very low. It should be noted that, there were no more than three studies reporting data on these there allergic conditions, and the pooled sample size is relatively small. Future studies revealing the correlation between tic disorders and these allergic conditions are needed to confirm this finding.

### 4.2. Correlation between Td and allergic conditions in immune mechanism

Previous studies revealed that interleukin-12 and tumor necrosis factor alpha concentrations at baseline were elevated in TS compared with control subjects (18, p. 667–73). Both of these markers were further increased during periods of symptom exacerbation. Similar findings have been reported in allergic rhinitis, allergic conjunctivitis and asthma, where factors such as TNF-Α and IL-5 play an important role in the pathogenesis of Allergic Rhinitis (19, p. 1139–1149). TNF-α monoclonal antibody significantly improves nasal symptoms in AR mice (20, p. 517–523). Patients with allergic conjunctivitis demonstrate an immunological dysregulation, characterized by the low expression of IL-10 and an inverted tear IL-10/TNF-α ratio (21). A systematic review concluded that TNF-α and IL-6 may influence the risk of asthma (22). The above findings suggest that tic disorders and a variety of allergic conditions both show abnormalities in inflammatory indicators such as interleukins and tumor necrosis factor, and that both may have similar inflammatory responses.

Multiplein studies demonstrate that the abnormality of T cell may be associated with the pathogenesis of TD in children (23, p. 519–523; 24). Some previous report had shown infection-induced immune mechanisms may also work in children with tic disorders and allergic conditions. Infection of pathogens such as cytomegalovirus, mycoplasma pneumoniae and streptococcus can not only cause tic disorder or deterioration, but also cause asthma attack, which may be related to immune dysfunction (25, p. e10; 26, p. 281–288; 27, p. 2035–2038; 28, p. 104893). Both immune dysregulation due to infectious diseases and inflammatory responses due to allergic conditions have a strong correlation with the onset or exacerbation of tic disorders, which warrants further research in the pathogenesis and clinical treatment of tic disorders.

### 4.3. Correlation between Td and allergic conditions in neuroimmunomodulator mechanism

Eye and nose symptoms such as blinking, wrinkling and throat clearing are often the initial manifestations of children with tic disorder, and these symptoms often run through the disease. At the beginning of the disease, children with TD are often misdiagnosed as allergic rhinitis and conjunctivitis. Apart from the possible correlation in immune mechanism, are the three diseases also related in terms of neuroregulation? It has been reported that the immune system directly or indirectly triggers the activation of peripheral neurons through inflammatory mediators such as cytokines, histamine and nerve growth factor (NGF) (29, p. 261–270; 30). This immune-nerve communication participates in nasal mucosal hyperresponsiveness, while the nervous system, including sensory nerve, sympathetic nerve and parasympathetic nerve, communicates directly with immune cells by releasing neuropeptides (SP, CGRP, VIP, etc.) and neurotransmitters (Ach, NE) (31, p. 6075–80; 32, p. 658; 33, p. 1085–93). An animal experiment had shown knockdown of neurokinin-1 receptor (NK-1R) expression decreased allergic inflammation in nasal mucosal tissues and alleviated the allergic rhinitis symptoms (34, p. 903–10). From the point of view of the developmental origin of nervous system, eye and nose, they all originated from the ectoderm of embryo. However, further studies are needed to determine the correlation between TD and allergic rhinitis and conjunctivitis in the pathogenesis and symptoms of neuroimmunomodulator mechanism. All of the above studies suggest that there may be some association between the nervous system and the immune system, which is consistent with our current findings that the neurological disease tic disorders may be associated with most allergic conditions. We hope to continue to investigate the mechanisms of tic disorders associated with allergic conditions in the future to try to provide ideas for the association between the nervous system and the immune system.

### 4.4. Strengths

Among previous investigations of the association between tic disorders and allergic conditions, there have been some differences or even contradictions between the results, and there have been some common problems, such as small sample sizes and single-center studies. In this review, through methods of systematic evaluation and meta-analysis, we combined the previous research results, generated a relatively large sample size, and determined the association between tic disorders and various allergic conditions. The strengths of this review include the comprehensive search in seven Chinese and English databases. Second, we used the modified Cochrane RoB tool—which excluded the “unclear” category—to evaluate the risk of bias. Additionally, we used GRADE as a tool to evaluate the certainty of evidence. The evidence profile findings identified four allergic conditions—allergic rhinitis, allergic conjunctivitis, eczema and food allergy—that were statistically associated with tic disorders (effect size: OR >2). Moreover, evidence certainty regarding the association between tic disorders and allergic rhinitis, eczema, food allergy was rated as moderate.

### 4.5. Limitations

It should be pointed out that, this study also had some limitations. First, in the analysis of urticaria, there were too few studies included, and the sample size was too small for the results to be representative. Second, we can only comment on the association between tic disorders and allergies but not the causal relationship or pathomechanism. Third, the studies included in this study were either from China or Turkey. Therefore, the diversity in the included populations is limited. Finally in the included studies, there were some allergic conditions diagnosed from subjective questionnaires, without specialist physical examination or auxiliary examination, meaning that our results are affected by recall bias. But as six of seven included studies showed low risk of bias, the associations observed in this meta-analysis could provide valuable directions for further prospective studies.

In conclusion, this review found associations between tic disorders and certain allergic conditions. However, the direction of causation and underlying mechanisms still need to be further studied. The association between tic disorders and allergies suggests that allergic conditions may be one of the causes of tic disorders. In clinical work, effective control of allergies may play a positive role in the prevention and control of tic disorders.

## Data Availability

The original contributions presented in the study are included in the article/**Supplementary Material**, further inquiries can be directed to the corresponding author.
