# Supplementary material for: The correlation between tic disorders and allergic conditions in children: A systematic review and meta-analysis of observational studies
Source: Front Pediatr. 2023 Mar 20;11:1064001. doi: 10.3389/fped.2023.1064001 (PMC10067604; doi:10.3389/fped.2023.1064001)
Supplement: Supplementary file 1 [file Datasheet1.docx]

**pubmed**

1.“Tic Disorders”[Mesh] OR “Tics”[Mesh] OR “Tourette Syndrome”[Mesh]

2."tourette*"[Title/Abstract] OR "tic"[Title/Abstract] OR "tics*"[Title/Abstract] OR "tic disorder*"[Title/Abstract]

3. #1 OR #2

4. “Asthma”[Mesh] OR “Rhinitis, Allergic”[Mesh] OR “Rhinitis, Allergic, Seasonal”[Mesh] OR “Conjunctivitis, Allergic”[Mesh] OR “Purpura, Schoenlein-Henoch”[Mesh] OR “Eczema”[Mesh] OR “Urticaria”[Mesh] OR “Atopic Dermatitides”[Mesh] OR “Food Hypersensitivity”[Mesh] OR “Drug Hypersensitivity”[Mesh]

5. ((((((((((intermittent[Title/Abstract]) OR (spring[Title/Abstract])) OR (summer[Title/Abstract])) OR (pollen[Title/Abstract])) OR (grass*[Title/Abstract])) OR (birch[Title/Abstract])) OR (ragweed[Title/Abstract])) OR (tree*[Title/Abstract])) OR (mugwort[Title/Abstract])) OR (willow[Title/Abstract])) AND ((rhinit*[Title/Abstract]) OR (Conjunctivit*[Title/Abstract]))

6. "asthma*"[Title/Abstract] OR "eczema*"[Title/Abstract] OR "atopic eczema"[Title/Abstract] OR "urticaria*"[Title/Abstract] OR "atopic dermatiti*"[Title/Abstract] OR "neurodermatiti*"[Title/Abstract] OR "hives"[Title/Abstract] OR "wheals"[Title/Abstract] OR "allergic*"[Title/Abstract]

7. #4 OR #5 OR #6

8.#3 AND #7

**Cochrane**

1.Mesh Tics (主题词)

2.(tourette*):ti,ab,kw OR (tic):ti,ab,kw OR (tics):ti,ab,kw

3.#1 OR #2

4.Mesh Rhinitis, Allergic(主题词)

5.Mesh Rhinitis, Allergic, Seasonal(主题词)

6.Mesh Conjunctivitis, Allergic(主题词)

7. Mesh Purpura (主题词)

8. Mesh Dermatitis (主题词)

9.Mesh Food Hypersensitivity(主题词)

10.Mesh Drug Hypersensitivity(主题词)

11.(asthma*):ti,ab,kw OR (Eczema):ti,ab,kw OR (Urticaria):ti,ab,kw OR (hives):ti,ab,kw OR (wheals):ti,ab,kw

12.#4 OR #5 OR #6 OR #7 OR #8 OR #9 OR #10 OR #11

13.#3 and #12

**Embase**

1.exp tic/

2.tourette*:ti,ab,kw OR tic:ti,ab,kw OR tics*:ti,ab,kw

3.#1 or #2

4. 'asthma'/exp OR 'allergic rhinitis'/exp OR 'allergic conjunctivitis'/exp OR 'anaphylactoid purpura'/exp OR 'eczema'/exp OR 'urticaria'/exp OR 'atopic dermatitis'/exp OR 'food allergy'/exp OR 'drug hypersensitivity'/exp

5. intermittent:ti,ab,kw OR spring:ti,ab,kw OR summer:ti,ab,kw OR pollen:ti,ab,kw OR grass*:ti,ab,kw OR birch:ti,ab,kw OR ragweed:ti,ab,kw OR tree*:ti,ab,kw OR mugwort:ti,ab,kw OR willow:ti,ab,kw

6. rhinit*:ti,ab,kw OR conjunctivit*:ti,ab,kw

7. #5 and #6

8. "asthma*"[Title/Abstract] OR "eczema*"[Title/Abstract] OR "atopic eczema"[Title/Abstract] OR "urticaria*"[Title/Abstract] OR "atopic dermatiti*"[Title/Abstract] OR "neurodermatiti*"[Title/Abstract] OR "hives"[Title/Abstract] OR "wheals"[Title/Abstract] OR "allergic*"[Title/Abstract]

9. #4 OR #7 OR #8

10. #3 AND #9

**CNKI**

(TKA = '抽搐性运动障碍' OR TKA = '抽搐障碍' OR TKA = '抽动' OR TKA = '多动秽语综合征' OR TKA = '图雷特综合征' OR TKA = '图雷特氏综合征') AND (TKA = '哮喘' OR TKA = '哮病' OR TKA = '过敏性鼻炎' OR TKA = '季节性鼻炎' OR TKA = '变应性鼻炎' OR TKA = '鼻鼽' OR TKA = '变应性结膜炎' OR TKA = '过敏性结膜炎' OR TKA = '结膜炎, 特应性' OR TKA = '结膜炎, 巨乳头状' OR TKA = '结膜炎, 春季' OR TKA = '角结膜炎, 春季' OR TKA = '卡他性结膜炎' OR TKA = '过敏性紫癜' OR TKA = '变应性紫癜' OR TKA = ' Henoch紫癜' OR TKA = ' Henoch-Schoenlein紫癜' OR TKA = '紫癜出血' OR TKA = ' 紫癜, 非血小板减少性' OR TKA = '类风湿性紫癜' OR TKA = ' Schoenlein-Henoch紫癜' OR TKA = '血管炎, 出血性' OR TKA = '湿疹' OR TKA = '皮炎, 湿疹性' OR TKA = '湿疮' OR TKA = '荨麻疹' OR TKA %= '鬼风疙瘩' OR TKA %= '瘾疹' OR TKA %= '赤白游风' OR TKA %= '特应性皮炎' OR TKA %= '湿疹, 特应性' OR TKA %= '湿疹, 婴儿' OR TKA %= '神经性皮炎, 特应性' OR TKA %= '神经性皮炎, 播散性' OR TKA %= '四弯风' OR TKA %= '食物过敏' OR TKA %= '食物变态反应' OR TKA %= '超敏反应, 食物' OR TKA %= '药物过敏' OR TKA %= '超敏反应, 药物' OR TKA %= '药疹' OR TKA %= '药物性皮炎')

**万方**

(主题:(“抽搐性运动障碍”) or 主题:(“抽搐障碍”) or 主题:(“抽动”) or 主题:(“多动秽语综合征”) or 主题:(“图雷特综合征”) or 主题:(“图雷特氏综合征”) ) and (主题:(“哮喘”) or 主题:(“哮病”) or 主题:(“季节性鼻炎”) or 主题:(“变应性鼻炎”) or 主题:(“鼻鼽”) or 主题:(“变应性结膜炎”) or 主题:(“过敏性结膜炎”) or 主题:(“结膜炎, 特应性”) or 主题:(“结膜炎, 巨乳头状”) or 主题:(“结膜炎, 春季”) or 主题:(“角结膜炎, 春季”) or 主题:(“卡他性结膜炎”) or 主题:(“过敏性紫癜”) or 主题:(“变应性紫癜”) or 主题:(“Henoch紫癜”) or 主题:(“Henoch-Schoenlein紫癜”) or 主题:(“紫癜出血”) or 主题:(“紫癜, 非血小板减少性”) or 主题:(“类风湿性紫癜”) or 主题:(“Schoenlein-Henoch紫癜”) or 主题:(“血管炎, 出血性”) or 主题:(“湿疹”) or 主题:(“皮炎, 湿疹性”) or 主题:(“湿疮”) or 主题:(“荨麻疹”) or 主题:(“鬼风疙瘩”) or 主题:(“瘾疹”) or 主题:(“赤白游风”) or 主题:(“特应性皮炎”) or 主题:(“湿疹, 特应性”) or 主题:(“湿疹, 婴儿”) or 主题:(“神经性皮炎, 特应性”) or 主题:(“四弯风”) or 主题:(“食物过敏”) or 主题:(“食物变态反应”) or 主题:(“超敏反应, 食物”) or 主题:(“药物过敏”) or 主题:(“超敏反应, 药物”) or 主题:(“药疹”) or 主题:(“药物性皮炎”))

**维普**

((M=(抽搐性运动障碍) OR M=(抽搐障碍) OR M=(抽动) OR M=(多动秽语综合征) OR M=(图雷特综合征) OR M=(图雷特氏综合征) ) AND (M=(哮喘) OR M=(哮病) OR M=(季节性鼻炎) OR M=(变应性鼻炎) OR M=(鼻鼽) OR M=(变应性结膜炎) OR M=(过敏性结膜炎) OR M=(结膜炎, 特应性) OR M=(结膜炎, 巨乳头状) OR M=(结膜炎, 春季) OR M=(角结膜炎, 春季) OR M=(卡他性结膜炎) OR M=(过敏性紫癜) OR M=(变应性紫癜) OR M=(Henoch紫癜) OR M=(Henoch-Schoenlein紫癜) OR M=(紫癜出血) OR M=(紫癜, 非血小板减少性) OR M=(类风湿性紫癜) OR M=(Schoenlein-Henoch紫癜) OR M=(血管炎, 出血性) OR M=(湿疹) OR M=(皮炎, 湿疹性) OR M=(湿疮) OR M=(荨麻疹) OR M=(鬼风疙瘩) OR M=(瘾疹) OR M=(赤白游风) OR M=(特应性皮炎) OR M=(湿疹, 特应性) OR M=(湿疹, 婴儿) OR M=(神经性皮炎, 特应性) OR M=(四弯风) OR M=(食物过敏) OR M=(食物变态反应) OR M=(超敏反应, 食物) OR M=(药物过敏) OR M=(超敏反应, 药物) OR M=(药疹) OR M=(药物性皮炎))) OR ((R=(抽搐性运动障碍) OR R=(抽搐障碍) OR R=(抽动) OR R=(多动秽语综合征) OR R=(图雷特综合征) OR R=(图雷特氏综合征) ) AND (R=(哮喘) OR R=(哮病) OR R=(季节性鼻炎) OR R=(变应性鼻炎) OR R=(鼻鼽) OR R=(变应性结膜炎) OR R=(过敏性结膜炎) OR R=(结膜炎, 特应性) OR R=(结膜炎, 巨乳头状) OR R=(结膜炎, 春季) OR R=(角结膜炎, 春季) OR R=(卡他性结膜炎) OR R=(过敏性紫癜) OR R=(变应性紫癜) OR R=(Henoch紫癜) OR R=(Henoch-Schoenlein紫癜) OR R=(紫癜出血) OR R=(紫癜, 非血小板减少性) OR R=(类风湿性紫癜) OR R=(Schoenlein-Henoch紫癜) OR R=(血管炎, 出血性) OR R=(湿疹) OR R=(皮炎, 湿疹性) OR R=(湿疮) OR R=(荨麻疹) OR R=(鬼风疙瘩) OR R=(瘾疹) OR R=(赤白游风) OR R=(特应性皮炎) OR R=(湿疹, 特应性) OR R=(湿疹, 婴儿) OR R=(神经性皮炎, 特应性) OR R=(四弯风) OR R=(食物过敏) OR R=(食物变态反应) OR R=(超敏反应, 食物) OR R=(药物过敏) OR R=(超敏反应, 药物) OR R=(药疹) OR R=(药物性皮炎)))

**CBM**

1. ("抽搐性运动障碍"[不加权:扩展]) OR "多动秽语综合征"[不加权:扩展]

2. "抽动"[常用字段:智能] OR "图雷特综合征"[常用字段:智能] OR "图雷特氏综合征"[常用字段:智能]

3. #1 or #2

4. ((((((("哮喘"[不加权:扩展]) OR "鼻炎, 过敏性"[不加权:扩展]) OR "结膜炎, 变应性"[不加权:扩展]) OR "紫癜, 过敏性"[不加权:扩展]) OR "湿疹"[不加权:扩展]) OR "荨麻疹"[不加权:扩展]) OR "超敏反应"[不加权:扩展]) OR "皮炎, 特应性"[不加权:扩展]

5. "哮病"[常用字段:智能] OR "鼻鼽"[常用字段:智能] OR "卡他性结膜炎"[常用字段:智能] OR "过敏性结膜炎"[常用字段:智能] OR "湿疮"[常用字段:智能] OR "瘾疹"[常用字段:智能] OR "赤白游风"[常用字段:智能] OR "四弯风"[常用字段:智能]

6. #4 or #5

7.#3 and #6
