# Supplementary material for: The correlation between tic disorders and allergic conditions in children: A systematic review and meta-analysis of observational studies
Source: Front Pediatr. 2023 Mar 20;11:1064001. doi: 10.3389/fped.2023.1064001 (PMC10067604; doi:10.3389/fped.2023.1064001)
Supplement: Supplementary file 2 [file Datasheet2.docx]

| Risk of bias of the included studies | | | | | |  |
| --- | --- | --- | --- | --- | --- | --- |
| Study | 1. Can we be confident in the assessment of exposure? | 2. Can we be confident that cases had developed the outcome of interest and controls had not? | 3. Were the cases (those who were exposed and developed the outcome of interest) properly selected? | 4. Were the controls (those who were exposed and did not develop the outcome of interest) properly selected? | 5. Were cases and controls matched according to important prognostic variables, or was statistical adjustment carried out for those variables | Risk of bias |
| Chang 2011 | DY | DY | DY | DY | DY | Low risk |
| Wu  2014 | PN | DY | PN | DN | PN | High risk |
| Yuce 2014 | DY | DY | DY | DY | PN | Low risk |
| Shen 2017 | PY | PN | PY | DY | PY | Low risk |
| Aksu 2020 | PN | DY | PY | DY | PY | Low risk |
| Chen 2020 | PY | DY | PY | PY | PY | Low risk |
| Chang  2022 | DY | DY | DY | DY | DY | Low risk |

DY: Definitely yes (low risk of bias); PY: Probably yes; PN: Probably no; DN: Definitely no (high risk of bias).
